# H.R.H. The Prince of Wales's Speech

**Published:** 1906-12-22

**Authors:** 


### H.R.H. THE PRINCE OF WALES'S SPEECH.

His Royal Highness the Prince of Wales, in moving the adoption of the reports and awards, said:—

In moving the adoption of the reports of the Executive and Distribution Committees I should like to take the opportunity of referring to certain matters of interest and importance connected with the Fund.

Through the death of Mrs. Lewis-Hill we shall receive £250,000, left to us by Mr. Lewis, her late husband. I am sure that this meeting will share my feelings of sincere regret at our loss through the death of this charitable lady, who was one of our most liberal supporters. For it must be remembered that, although that sum was left to Mrs. Lewis-Hill for her life, she contributed to the Fund during her lifetime the income of it—£10,000 a year. We shall now receive the capital sum, together with half the residue of Mr. Lewis's estate.

And there is another loss which we have to deplore, one whose name will never be forgotten as the true friend and the noble-minded benefactor of the sick and poor, Mr. George Herring. From him we also receive a considerable bequest in favour of the Fund.

These sums, together with the large gifts received in recent years, and legacies of £20,000 from Mr. Beit, £10,000 from Mr. John Nicholas, £6,700 from Mrs. Finnie, and a large amount to be received from the estate of the late Mr. Heigham, as well as a gift of £10,000 from Mr. and Mrs. Bischoffscheim, and an equal sum from an anonymous donor, will bring our capital up to a large figure.

You will all, I believe, agree that the time has come when a committee should be appointed in order to assist Lord Rothschild and Mr. H. C. Smith, who have hitherto so ably attended to the investment of our capital. With their approval I have asked and have been lucky enough to obtain the valuable assistance of Lord Revelstoke, Sir Ernest Cassel, and Mr. Fleming, who have joined that committee.

After careful consideration, acting on legal advice, I have, with the sanction of the King, determined to apply to Parliament for a short Act to incorporate the Fund and to place its administration upon a strictly legal basis. I much regret that, owing to the shortness of time at our disposal before the Bill had to be lodged, which happens to be this very day, and to avoid losing a whole year, the Bill could not be submitted to you as I should naturally have desired. Copies are now before you, and I trust that it will meet with your entire support and approval. I am sorry that time did not admit of my consulting you, gentlemen, as a whole, but I had the assistance and advice of those who are most closely connected with the matter, and I appointed a committee to confer with those responsible for the drafting of the Bill.

As soon as we obtain our necessary statutory powers it may be advisable to consider the constitution of the Council and of the committees, and take steps to ensure that their respective duties and responsibilities are more closely defined than they have hitherto been.

Though our capital is large, we still look and hope for more support from annual subscribers of small sums. And I cannot but think that many who have been treated in the London hospitals, or their friends, would, if the matter were suggested to them in the proper light, contribute something—no matter how small—as a thank-offering. I watch with the liveliest interest and pride the development of our great charity, but I should like to feel that it had the general support of the people of London; and this can only be proved by a constantly increasing number of annual subscribers.

As Sir Henry Burdett has just told us, the League of Mercy has again given us splendid support—£18,000, an increase of £3,000 upon the sum given last year. It is with much pleasure that I make this announcement, as it is a testimony of the steady growth and expansion of the work of the League. I know that these results are not attained without very considerable efforts, and I heartily congratulate the members of the League on the gratifying results of their labours.

I am glad that we have been able to increase the sum to be distributed this year to £111,000, which includes the £1,000 kindly entrusted to us by the London Parochial Charities for the Convalescent Homes. I hope that future distributions will never fall below this amount.

We have continued the issue of our Statistical Report on Hospital Expenditure and Prices, which began in 1904 with the sixteen large general hospitals. The value of Mr. Danvers Power's work in this respect cannot be too highly appreciated. It now includes forty-eight hospitals. The sixteen hospitals reported on in 1904 showed a large reduction of expenditure last year, the value of which, taking into consideration the increase in the number of beds, is equal to a saving of about £20,000. When the other hospitals have had time to take advantage of the information which our reports provide, we hope this satisfactory saving will be largely increased.

I desire to acknowledge the ready way in which many of the hospitals have seconded our efforts by the appointment of Economy Committees.

Let me take this opportunity of thanking Mr. J. G. Griffiths for his valuable report, made at my request, on the uniform system of hospital accounts, on which our statistics are based. My thanks are also due to the Committee of Hospital Secretaries, presided over by Mr. Ryan. They took Mr. Griffiths's recommendations into consideration, with the final result that the Hospital Sunday and Saturday Funds have agreed with us on a revised system. This will come into force on January 1 next, and I trust will be of benefit to all concerned in this important work of regulating hospital expenditure and thus saving money, which is the same thing as gaining it, or even better.

To all the honorary officials of the Fund and those who have served on the Council and various committees I offer my warmest thanks. I cannot exaggerate what we owe to our honorary secretaries—they are unsparing in the valuable time and services which they, I fear often at personal inconvenience, cheerfully place at my disposal and that of the Fund. I am glad to think that their labours will be now to some extent lightened by the appointment of Mr. Maynard as Secretary of the Fund. But besides specifying the honorary officials of the Fund, I am anxious to assure every member of the Council and committees how deeply I realise and appreciate the fact that it is by the gift of such of their valuable time and services as they are able to offer that this great and ever-increasing work of charity is administered, and I venture to say well and successfully administered; and to them all I offer my expressions of heartfelt gratitude.

The Lord Mayor (Sir W. P. Treloar), in seconding the adoption of the report, said he was glad of the opportunity of saying, as Lord Mayor, that he was confident the Corporation of the City of London would, as heretofore, continue to do all they could to support the Fund.

The resolution was then put and carried unanimously.

On the motion of the Bishop of London it was resolved that the cheques for the grants should be posted on December 20.

### Incorporation of the Fund.

Mr. Stuart-Wortley moved that the Council approves of the Bill to incorporate the Fund. He explained the provisions of the Bill, which provides that the Fund shall be a body corporate, with power to hold land, and generally have also the rights of a body corporate. The Bill gave full power to the President, who would be nominated by the Sovereign, and provided for the scope of the Fund's work, indemnifying all persons who have acted in good faith in carrying out the trusts of the Fund hitherto.

Sir Joseph Dimsdale seconded.

Lord Rothschild said that the Trustees had had legal advice to the effect that, unless otherwise provided by donors, gifts of money to the Fund must be invested in trust funds. A large sum of £100,000 recently given to the Fund in bonds, which had been paid off, had accordingly been so invested, and that had given rise to some dissatisfaction. A Finance Committee could not deal with these matters until given the power asked for in the Bill.

Sir Henry Burdett thought that to include dispensaries and medical missions might extend the operations of the Fund too much.

Mr. Danvers Power explained that the fullest permissive powers had been taken, but that the President would define the limits of action from time to time.

Sir William Collins said he welcomed the proposed incorporation of the Fund, because, as his Royal Highness had foreshadowed, it would be followed by regularising and defining the duties of the various committees of the Fund. He had not been able to read the Bill through since he entered the room, but it occurred to him that Clauses 5 and 8 might give rise to discussion in the House of Commons and might necessitate some give-and-take. As a member of the Lower House, while voting for the approval of the Bill at that meeting, he desired to reserve some liberty of action when the matter got into Committee in the Commons.

The motion was carried unanimously.

### Votes of Thanks.

Mr. Evan Spicer (Chairman of the County Council), in moving a vote of thanks to his Royal Highness for presiding, congratulated him upon the splendid results which had been attained.

Sir William Collins seconded, and took the opportunity as honorary secretary of the League of Mercy of thanking the Prince and Princess of Wales for their splendid hospitality in giving the garden-party each year, and for what his Royal Highness had said about the League, which would be an encouragement to all its members. (Hear, hear.)

The vote being carried by acclamation, his Royal Highness said :—

My Lords and Gentlemen,—I thank the Chairman of the County Council and Sir William Collins for the kind vote of thanks which they proposed and seconded. I have always felt that the King conferred a great privilege upon me when he made me President of this Fund, which he had himself initiated. The King desires me to offer you his congratulations on the gratifying results of the past year, and to tell you how much he appreciates the energy and self-sacrifice which have been displayed by those in whose hands has been placed the administration of the Fund. I can only say that it has given me great pleasure to meet you here again in my house, especially as last year, owing to my absence in India, I was unable to do so.

The proceedings then terminated.

